# Connectivity between the superior colliculus and the amygdala in humans and macaque monkeys: virtual dissection with probabilistic DTI tractography

**DOI:** 10.1152/jn.01016.2014

**Published:** 2015-07-29

**Authors:** Robert D. Rafal, Kristin Koller, Janet H. Bultitude, Paul Mullins, Robert Ward, Anna S. Mitchell, Andrew H. Bell

**Affiliations:** ^1^Wolfson Centre for Clinical and Cognitive Neuroscience, School of Psychology, Bangor University, Bangor, Gwynedd, United Kingdom;; ^2^Centre for Functional Magnetic Resonance Imaging of the Brain, Nuffield Department of Clinical Neurosciences, University of Oxford, Oxford, United Kingdom;; ^3^Department of Experimental Psychology, University of Oxford, Oxford, United Kingdom; and; ^4^MRC Cognition and Brain Sciences Unit, Cambridge, United Kingdom

**Keywords:** threat, DTI tractography, superior colliculus, pulvinar, amygdala

## Abstract

It has been suggested that some cortically blind patients can process the emotional valence of visual stimuli via a fast, subcortical pathway from the superior colliculus (SC) that reaches the amygdala via the pulvinar. We provide in vivo evidence for connectivity between the SC and the amygdala via the pulvinar in both humans and rhesus macaques. Probabilistic diffusion tensor imaging tractography revealed a streamlined path that passes dorsolaterally through the pulvinar before arcing rostrally to traverse above the temporal horn of the lateral ventricle and connect to the lateral amygdala. To obviate artifactual connectivity with crossing fibers of the stria terminalis, the stria was also dissected. The putative streamline between the SC and amygdala traverses above the temporal horn dorsal to the stria terminalis and is positioned medial to it in humans and lateral to it in monkeys. The topography of the streamline was examined in relation to lesion anatomy in five patients who had previously participated in behavioral experiments studying the processing of emotionally valenced visual stimuli. The pulvinar lesion interrupted the streamline in two patients who had exhibited contralesional processing deficits and spared the streamline in three patients who had no deficit. Although not definitive, this evidence supports the existence of a subcortical pathway linking the SC with the amygdala in primates. It also provides a necessary bridge between behavioral data obtained in future studies of neurological patients, and any forthcoming evidence from more invasive techniques, such as anatomical tracing studies and electrophysiological investigations only possible in nonhuman species.

the amygdala is critically involved in responding to threat and in the ability to extract emotional information from faces that is needed for successful adaptation in a social environment (Adolphs 2008; Adolphs and Spezio 2006). The main pathway for the transmission of visual information from the retina to the amygdala passes through striate and extrastriate cortex. Morris and colleagues (de Gelder et al. 2005; Morris et al. 1999), however, postulated the existence of a second, subcortical route from the superior colliculus (SC) to the amygdala via the pulvinar nucleus of the thalamus. This putative fast, subcortical pathway would presumably allow for the rapid, unconscious, processing of emotional stimuli (see Tamietto and de Gelder 2010 for review).

Nevertheless, the case for a fast, unconscious subcortical pathway from the SC to the amygdala via the thalamus remains contentious (de Gelder et al. 2011; Pessoa and Adolphs 2010; Tamietto and de Gelder 2010), and gaining understanding about three outstanding questions may help resolve this controversy. *1*) Is there anatomical evidence that such a pathway exists in the primate brain? *2*) Is the processing mediated by this putative pathway unconscious? *3*) Is it a faster route than circuits transmitting affective signals to the cerebral cortex?

The current investigation addresses the first of these questions. It demonstrates, using probabilistic tractography, connectivity between the SC and amygdala, passing through the medial pulvinar, in humans and macaque monkeys. The trajectory of this connection from the thalamus traverses above the temporal horn of the lateral ventricle to connect to the lateral amygdala. We further demonstrate the trajectory of this pathway relative to the stria terminalis.

Tamietto et al. (2012) have recently used in vivo tractography to demonstrate connectivity between the SC and amygdala passing through the pulvinar in humans. They also provided evidence for a visual function of the pathway, and a role for it in mediating blindsight, by showing that the connections in the hemisphere with lesioned striate cortex were stronger in a hemianopic patient with blindsight than in non-hemianopic controls without brain damage. Here, we expand on these results by demonstrating this pathway as an isolated streamline and showing for the first time in any species its trajectory between the thalamus and the amygdala. We supplement evidence in humans with that from nonhuman primates (*Rhesus macaques*), thereby facilitating comparisons between data obtained using probabilistic tractography and invasive anatomical tracing studies. We also demonstrate the potential application of tractography in patients with pulvinar lesions to examine the role of the pathway in processing emotionally valenced visual stimuli.

## METHODS

### Scanning Protocols

#### Human.

Initially, eight neurologically healthy young adults (5 women and 3 men) were recruited from Bangor University students and research staff (“first group”). To replicate the findings in the first group in a larger sample, we collected diffusion-weighted data with more diffusion directions, higher b-values and larger voxel size (to achieve a greater signal-to-noise ratio) in an additional 12 (6 women and 6 men) human participants (“second group”). Oral and written, informed consent from all 20 participants was obtained under a protocol approved by the School of Psychology Ethics Committee. Participants received course credit or £10 for participation.

All neuroimaging data from human participants were collected using a Philips Achieva 3T Scanner at Bangor University. In the first group (*n* = 8), diffusion-weighted echo-planar (DW-EPI) images were collected at 1.5 × 1.5 × 1.5 mm resolution. The imaging parameters consisted of b values = 0 (four volumes) and 800, 32 isotropically distributed diffusion-encoding directions, repetition time (TR) = 2.0 s, and echo time (TE) = 35 ms. In the second group (*n* = 12), DW-EPI images were collected at 2 × 2 × 2 mm resolution using the following parameters: b values = 0 (four volumes) and 2,000, 61 distributed diffusion directions, TR = 2 s, and TE = 35 ms. Diffusion-weighted images were converted to NIFTI format for offline analysis using the FSL-FDT software suite (Andersson et al. 2003; Jenkinson et al. 2012; Smith et al. 2004; Woolrich et al. 2009) and FDT toolbox (Behrens et al. 2003, 2007) (http://fsl.fmrib.ox.ac.uk/fsl/fslwiki/). Diffusion-weighted images were corrected for eddy currents and head motion using affine registration to the first b-zero volume, and the diffusion parameters were estimated by running the Markov Chain Monte Carlo sampling method.

T1-weighted, high-resolution (0.7 mm isotropic voxels) structural images were collected for all 20 participants using an MPRAGE (magnetization prepared gradient echo) sequence. The T1-weighted structural images were aligned to the individual's diffusion-weighted images, and masks were drawn around the SC, pulvinar, and amygdala, as described below.

#### Monkey.

Eight adult male monkeys (*Macaca mulatta*, 6–11 kg), purpose bred in the United Kingdom, were used in this study. All animal procedures were conducted in accordance with the United Kingdom Animals (Scientific Procedures) Act (1986) and approved by the University of Oxford local ethical review panel and the UK Home Office Animal Inspectorate. Animals were socially-housed (between 4 and 8 per group) with varying forms of environment enrichment. Animals were given ad libitum access to water, and regular health and welfare assessments were performed by animal care and veterinary staff, which included formalized behavioral monitoring.

All nonhuman primate imaging data were collected under general anesthesia. Data were collected on a 3T scanner using a custom-made 4-channel phased array coil (H. Kolster, MRI Coil Laboratory, Laboratory voor Neuro en Psychofysiologie, KU Leuven). Procedures for inducing and maintaining general anesthesia and the positioning of the monkeys in the scanner are similar to those described previously (Mars et al. 2011). DW-EPI images were collected at 1 × 1 × 1 mm resolution. The imaging parameters consisted of b values = 1 and 1,000, 60 isotropically distributed diffusion-encoding directions, TR = 8.3 s, and TE = 102 ms, with alternative phase-encoding directions (anterior-posterior, posterior-anterior). Six averages (each average consisting of 60 diffusion-encoding directions and 11 b = 0 images) were collected in two alternating phase-encoding directions within a single scan session, for a total of 12 complete datasets. The alternating phase-encoded datasets were later combined using the EPI distortion correction tool “Top-Up” (Andersson et al. 2003; Smith et al. 2004), which is part of the FSL analysis package to correct for susceptibility field distortions along the phase-encoding direction.

T1-weighted, high-resolution (0.5-mm isotropic voxels) structural images were collected using an MPRAGE (TR = 2.5 s, TE = 4.01 ms, 3–5 averages) sequence. Coil inhomogeneity for the anatomical images was corrected for by dividing the MPRAGE data by a lower resolution MPRAGE sequence (1 × 1 × 1 mm) that did not include an inversion recovery pulse (parameters: TR = 2.5 s, TE = 3.48 ms) (Van de Moortele et al. 2009). Data obtained in nonhuman primates were prepared for probabilistic tractography using the same procedures as were described for humans.

### Probabilistic Tractography

#### Virtual dissection of the connections between the SC and the amygdala in the human and monkey brain.

**Fig. 1.**
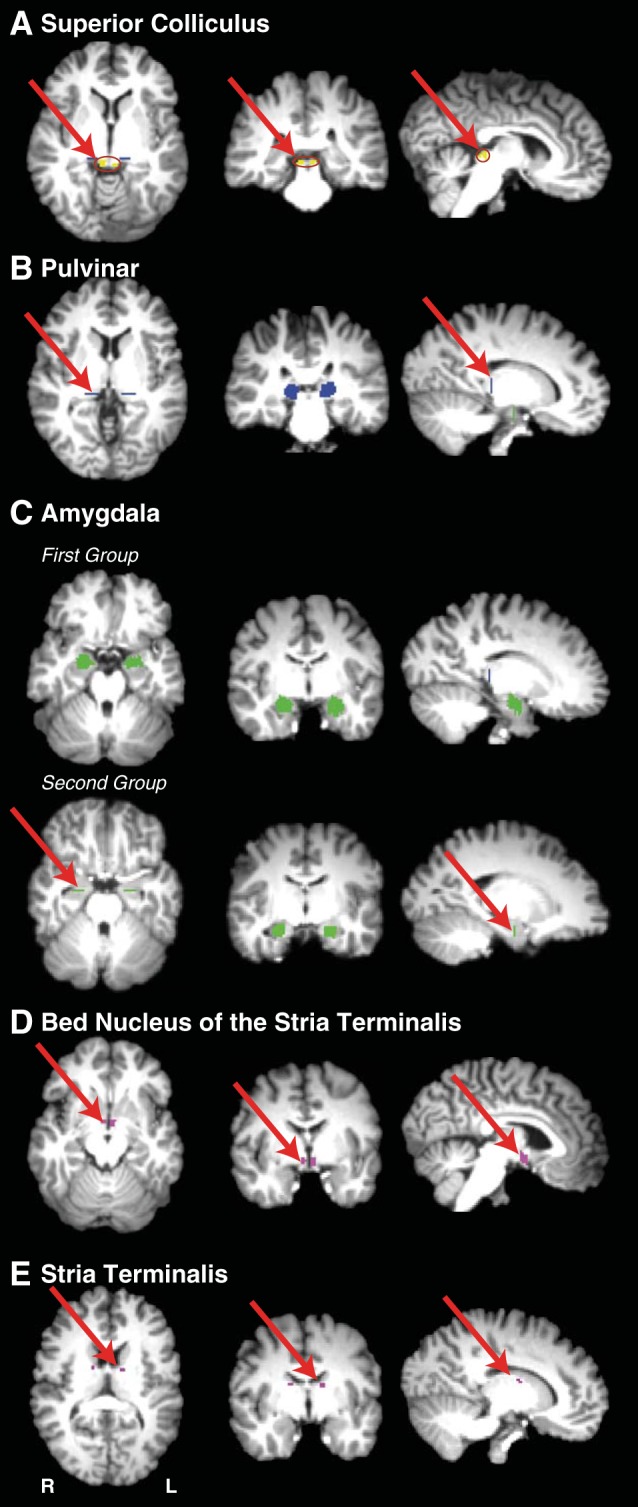
Masks used for the virtual dissection of possible connectivity between the superior colliculus (SC) and the amygdala, and for the dissection of the stria terminalis in the first group of human participants. *Left*, axial sections; *middle*, coronal sections; *right*, sagittal sections. *A*: SC masks (shown in yellow). *B*: pulvinar masks (shown in blue). *C*: amygdala masks for first and second groups (shown in green). *D*: bed nucleus of the stria terminalis masks (shown in magenta). *E*: stria terminalis waypoint mask subjacent to the frontal horn of the lateral ventricle between the head of the caudate nucleus and the thalamus.

Probabilistic tractography was implemented using ProbtrackX from the FSL FDT toolbox (curvature threshold = 0.2, number of samples = 5,000). Tractography between the SC and the amygdala was performed in both hemispheres of each individual human and monkey's diffusion-weighted images using the subject-specific SC, pulvinar, and amygdala masks (see Figs. 1 and 4 for sample masks for human and monkey brains, respectively). To evaluate the robustness of the observed streamlines, a pulvinar waypoint mask was used in the first group of human participants (so that only streamlines passing from the SC to the amygdala via the pulvinar would be revealed), but not with the second group of human participants. Both methods were contrasted in the monkey data. Omitting the pulvinar waypoint mask had minimal effect in either species (e.g., compare Fig. 3, *A* and *B*).

**Fig. 2.**
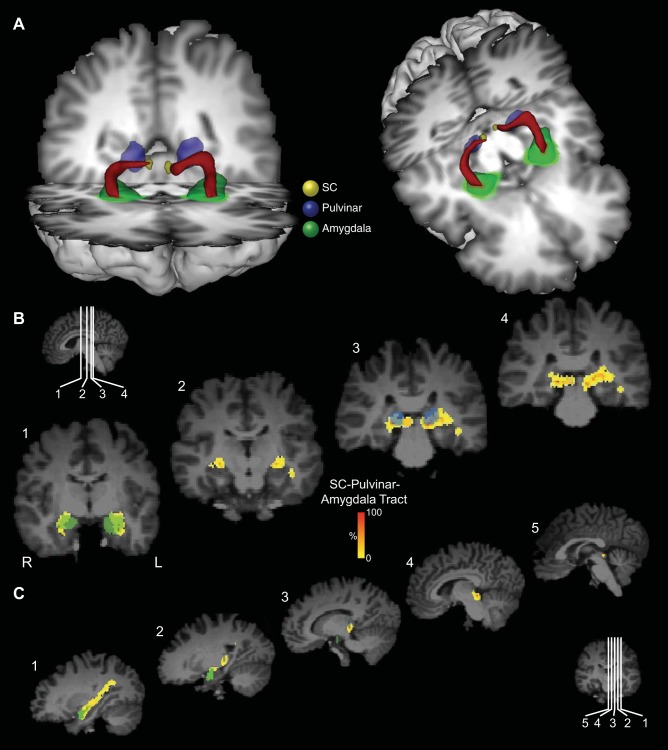
Probabilistic tractography between the SC and the amygdala via the pulvinar in one representative participant from the first group. *A*: three-dimensional (3D) reconstructions of the tract (shown in red) linking the SC (shown in yellow) and the amygdala (shown in green) via the pulvinar (shown in blue). Tracts have been thresholded by 10% (see methods for details). The individual structures and tracts in the 3D reconstructions have been dilated by 2–4 mm for visualization purposes only. Coronal (*B*) and sagittal (*C*) sections show the location of the tract relative to the amygdala (green), pulvinar (blue), and SC (yellow). The probabilistic data are presented unthresholded (and actual size) as a percentage of the total number of traces linking the SC and amygdala (that pass through the pulvinar).

The value of each voxel of the resulting streamline represented the total number of traces passing through that voxel. We thresholded each voxel so that only those voxels that contained at least 10% of the maximum number of traces found in any voxel remained. A very recent study that directly compared diffusion tensor imaging (DTI) tractography with tracers in monkeys has reported that a threshold of 10% is optimal to most reliably reflect the anatomy of tractography compared with tracers (Azadbakht et al. 2015). The thresholded tracts were projected onto three-dimensional (3D) reconstructions of the T1-weighted structural images (e.g., Fig. 2).

In the case of some human participants in the first group (*n* = 3/8), in addition to the putative SC-pulvinar-amygdala tract, probabilistic tractography produced additional streamlines that caused the SC-pulvinar-amygdala tract to partially divert into the stria terminalis and/or optic tract. We, therefore, used an additional coronal exclusion mask around the medial diencephalon in these participants to isolate the SC-pulvinar-amygdala tract. For uniformity, an exclusion mask was subsequently used for all participants in the second group of human participants.

All masks for human participants were drawn manually using anatomical landmarks. In the first group of human participants (*n* = 8), the SC was used as a seed mask and pulvinar and amygdala as waypoint masks, with the amygdala mask also used as a termination mask. The pulvinar waypoint masks were drawn on a single coronal slice, just anterior to the SC mask, which encompassed the gray matter of the pulvinar. The amygdala masks were drawn on a series of sagittal slices using the gray-white matter junction at the border of the amygdala as a landmark, taking care to exclude extraneous white matter beyond this border. This mask was continued into the gray matter of the amygdala extending over the dorsal tip of the temporal horn, but taking care to exclude gray matter of the hippocampus below the temporal horn (Fig. 1*C*). Masks were drawn by one investigator and inspected for accuracy by another.

In the second group of human participants (*n* = 12), the amygdala mask was drawn on a single coronal slice at the posterior border of the amygdala (Fig. 1*C*). The amygdala was used as a seed mask, and the pulvinar and SC as waypoint masks, with the SC also used as a termination mask. The order of the masks (SC/amygdala as seed vs. termination) and using masks composed of single slices vs. using three-dimensional masks had little qualitative effect on the results (confirmed by directly comparing the different approaches in the majority of human and monkey datasets, see below).

In the case of the monkey subjects, masks were drawn around the SC, pulvinar, and amygdala in all three dimensions based on atlas-based anatomical landmarks (Saleem and Logothetis 2006) and manually corrected to avoid extraneous white matter (see Figs. 4–6).

#### Dissection of the stria terminalis in the human brain.

**Fig. 3.**
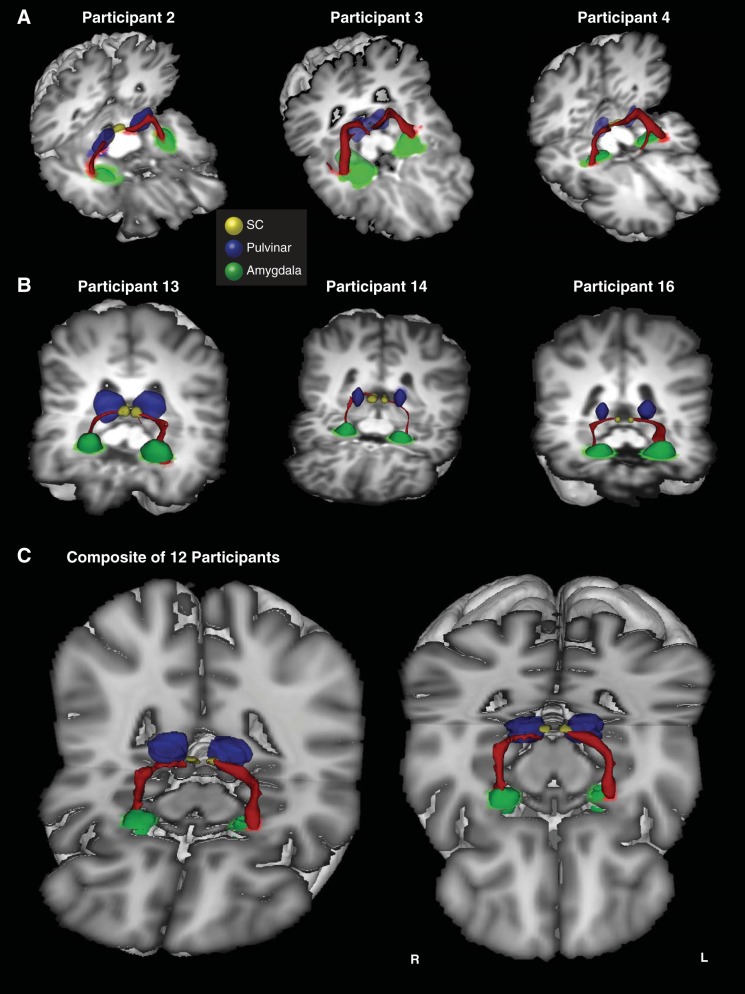
3D reconstructions of the SC-pulvinar-amygdala tract (shown in red) in 3 additional representative participants from the first (*A*) and second (*B*) groups. Tracts have been thresholded by 10% (see methods for details). The individual structures and tracts in the 3D reconstructions have been dilated by 2–6 mm for visualization purposes only. *C*: composite tract of all 12 participants from the second group (higher resolution). The composite tract has been thresholded such that only voxels that were identified in at least 75% of participants are shown.

As will be shown, the trajectory of the connections between the SC and the amygdala was found to traverse above the temporal horn of the lateral ventricle. The principle amygdalo-fugal projection, the stria terminalis, also traverses above the temporal horn of the lateral ventricle, and its topography has been demonstrated with DTI tractography (Avery et al. 2014; Kwon et al. 2011). Aggleton and Mishkin (1984) reported that some amygdalo-fugal projections to the thalamus might run in the stria terminalis. Although amygdala efferents to the thalamus project to midline thalamic nuclei and do not project to the pulvinar (Aggleton and Mishkin 1984), it was nevertheless important to identify the trajectory of the stria terminalis in our study for two reasons. First, it was necessary to show that the connections found between the SC and the amygdala were not an artifact resulting from crossing of fibers connected to the pulvinar converging with the stria terminalis. Second, in several participants, the connections between the SC and the amygdala shared voxels with the stria terminalis, and the stria terminalis was demonstrated in the same dissection as the connections between the SC and the amygdala, unless an exclusion mask was applied. Therefore, in each hemisphere of each human participant in the first group, a second virtual dissection of the SC-pulvinar-amygdala connections was performed that used the unthresholded stria-terminalis streamline as an exclusion mask to exclude voxels shared with the stria terminalis (e.g., Fig. 3).

The stria terminalis dissection was achieved using the same mask of the amygdala as seed, waypoint masks along the trajectory of the stria terminalis (between the caudate and anterior thalamic nuclei subjacent to the frontal horn of the lateral ventricle) and another in the region of bed nucleus of the stria terminalis (near the anterior commissure just lateral to the fornix where it traverses the hypothalamus) (see Fig. 1).

## RESULTS

### Connections Between the SC and the Amygdala in the Human and Monkey Brains

Connections between the SC and the amygdala were demonstrated in both hemispheres of 7 out of the 8 human participants in the first group, and in both hemispheres of all 12 participants in the second group. In the first group of participants, a single, isolated streamline was generated in both hemispheres without thresholding and without the use of an exclusion mask in five individuals. In the three remaining participants, a coronal exclusion mask through the diencephalon and optic tracts was necessary to isolate the streamline from also detouring into the optic tracts or stria terminalis. An exclusion mask was subsequently used in all 12 participants in the second group as a matter of course.

A 3D reconstruction of this streamline is shown for one representative human in Fig. 2*A* and for an additional six representative participants in Fig. 3 (Fig. 3*A*: first group; Fig. 3*B*: second group; Fig. 3*C*: composite of all data from the second group). Comparing the streamlines generated in the participants in the first group (Fig. 3*A*) with those from the second group (Fig. 3*B*) confirms that reversing starting and terminations masks, using 2D single-slice vs. 3D volume masks, or omitting the pulvinar mask (see methods) had no effect on the topography of the streamline.

The topography of the connections and anatomical relationships to other structures can be visualized by following the connections in a series of coronal (Fig. 2*B*) and sagittal (Fig. 2*C*) sections. The pathway initially ascends dorsally from the SC and then, in most hemispheres, continues to ascend postero-laterally to the pole of the medial pulvinar before turning ventro-laterally to a position above the temporal horn of the lateral ventricle. It then proceeds rostrally above the temporal horn and crosses from medial to lateral and then, terminally, turns medially to connect with the lateral amygdala. Animated versions of the streamlines are included in Supplemental Videos S1 and S2 (Supplemental material for this article is available online at the Journal website).

**Fig. 4.**
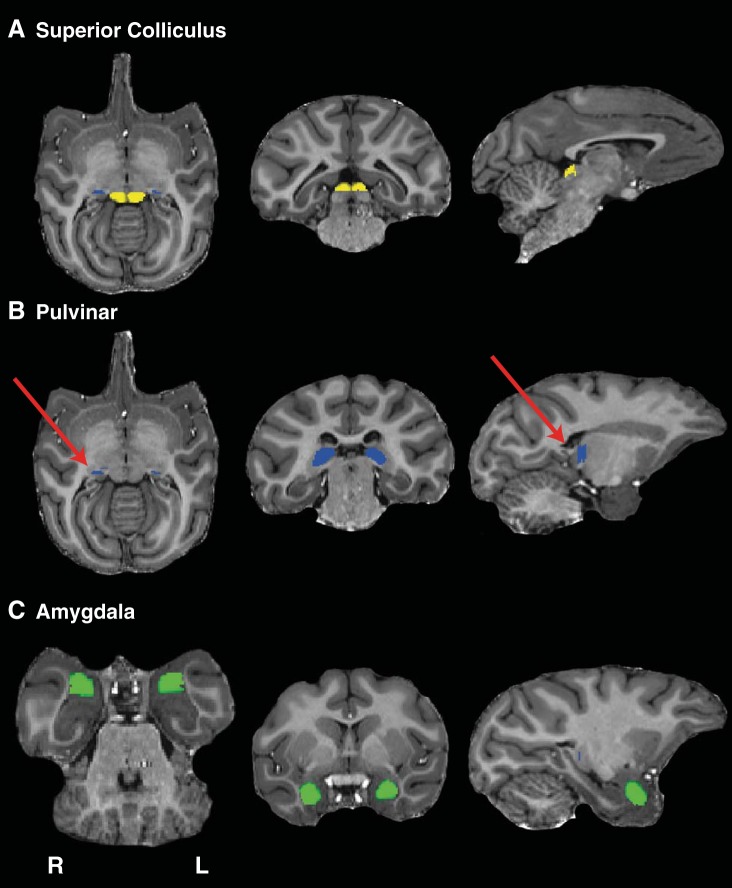
Masks used for the dissection of possible connectivity between the SC and the amygdala in the monkey brain. *Left*, axial sections; *middle*, coronal sections; *right*, sagittal sections. *A*: SC masks (shown in yellow). *B*: pulvinar masks (shown in blue). *C*: amygdala masks (shown in green).

**Fig. 5.**
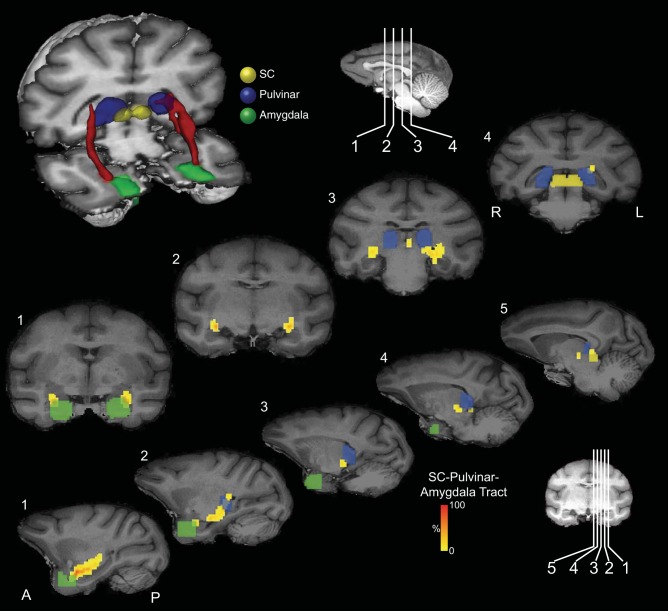
Probabilistic tractography between the SC and the amygdala via the pulvinar in the monkey brain. *Top*: 3D reconstructions of the tract (shown in red) linking the SC (shown in yellow) and the amygdala (shown in green) via the pulvinar (shown in blue). Coronal (*middle*) and sagittal (*bottom*) sections show the location of the tract relative to the amygdala (green), pulvinar (blue), and SC (yellow). The probabilistic data are presented unthresholded as a percentage of the total number of traces linking the SC and amygdala (that pass through the pulvinar).

**Fig. 6.**
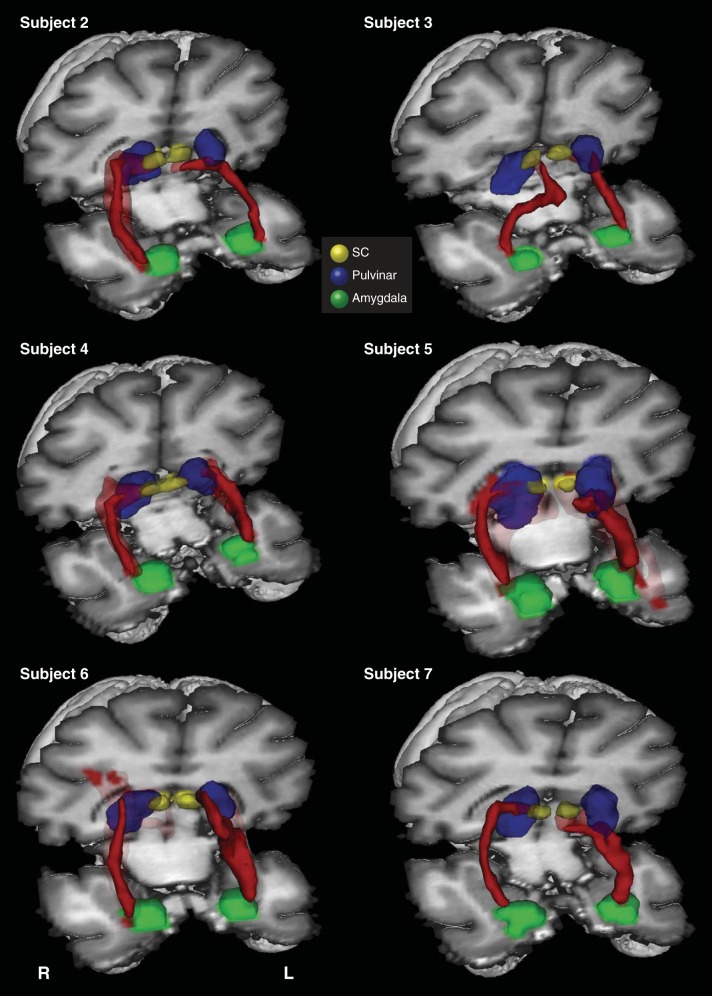
3D reconstructions of the SC-pulvinar-amygdala tract (shown in red) in the remaining 6 monkey subjects (in whom the tract was identified). Tracts have been thresholded by 10% (see methods for details). Unthresholded data are shown as transparency.

Tractography in macaque monkeys was implemented using masks of the SC, pulvinar, and amygdala, analogous to those used in human participants (see Fig. 4 for sample masks for the monkey brain). Connections between the SC and the amygdala were demonstrated in both hemispheres of seven of the eight subjects. Figure 5 shows a 3D reconstruction of these tracts in one representative subject, accompanied by coronal and sagittal sections. Figure 6 shows 3D reconstructions for the remaining six subjects in whom a tract was identified. In the majority of cases, the trajectory of the connections was similar to that seen in humans. The pathway ascends from the SC to the medial pole of the pulvinar and descends as it moves laterally to a position above the temporal horn of the lateral ventricle, which it traverses above to the lateral aspect of the amygdala.

### Analyses of the Variability of the Connection between the SC and the Amygdala

To obtain a statistical estimate of the intersubject variability in the trajectory of the streamlines, several additional analyses were performed. In the case of the human data, these steps were restricted to data obtained from the second group of human participants to take advantage of the increased sensitivity of these data.

Similar to Tamietto et al. (2012), tractography was implemented twice in each hemisphere with the SC used as the seed mask in one dissection and the amygdala in the other (no pulvinar waypoint mask was included). The two streamlines were combined by binarizing the streamlines, adding them together, and thresholding the result to produce an overlapping streamline showing only voxels in which a continuous common streamline connected SC-amygdala and amygdala-SC. Using this method, a continuous streamline was observed in the left hemisphere of nine participants and in the right hemisphere of eight participants.

This approach was less successful in monkeys. A continuous connection between the SC and the amygdala formed from overlapping streamlines was only observed in three of eight monkeys (both hemispheres). We, therefore, opted to analyze the single streamlines linking the SC and the amygdala (no pulvinar mask).

Overlapping the streamlines in each hemisphere and participant in this way affords the opportunity to more directly evaluate the variability in streamline topography across individuals. Averaging the two opposing streamlines in each participant reduced the noise and gave a better estimate of the most probable topography of the connections. We used these averaged streamlines to estimate the variability in topography (see below). In cases where an overlapping streamline was not generated by adding the two opposing streamlines (which occurred in 7 of the 24 hemispheres in the human dataset and in all hemispheres in the monkey dataset), we used the single streamline that corresponded most strongly to the topography demonstrated by the population.

**Fig. 7.**
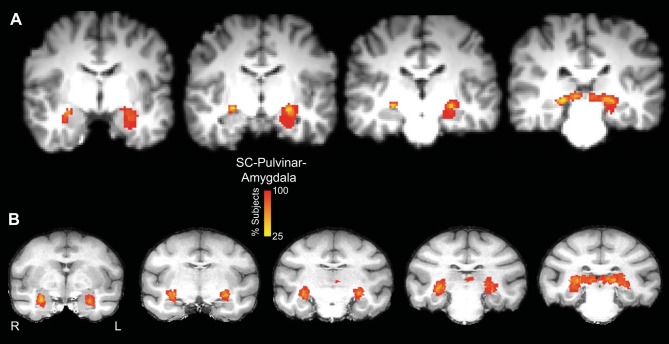
Overlap of binarized streamlines between the SC and the amygdala for all 12 participants (the second group) aligned to the Montreal Neurological Institute T1-weighted standard atlas in the coronal plane (*A*) and 8 monkeys (*B*). The color scale shows the proportion of participants whose streamline runs through each voxel.

Figure 7 shows the averaged streamlines for all 12 human participants (Fig. 7*A*), and all 8 monkey subjects (Fig. 7*B*), aligned to a single T1-weighted image for each species. The color scales in Fig. 7 represent the proportion of participants/subjects through which the streamline passed in each voxel. The variability seen in Fig. 7 reflects not only the variability in the trajectory of the streamlines generated in each individual, but also the variability in the size of the individual streamlines.

To obtain a statistical estimate of the intersubject variability in the trajectory of the streamlines, the “center of gravity” (CoG) of each streamline was computed in a series of coronal slices. In humans, we restricted this analysis to a segment of the streamline that traversed above the temporal horn, as this part of the streamline runs perpendicular to the coronal plane. In each of eight coronal slices covering this segment, the CoG for each participant's streamline in each hemisphere was computed in right-left (*x*) and superior-inferior (SI) (*z*) coordinates and averaged across all participants. Table 1 reports the variability (in the form of standard deviation) of the in-plane CoG across all human participants. Overall, there was little variability in the streamline across participants (at least in the segment of the connections traversing the temporal horn). In both *x* and *z* coordinates, the mean standard deviation of the CoG (averaged across the eight slices) across participants was 1–2 mm and thus near the anatomical resolution of the measurement.

**Table 1.** Standard deviation (in 2.0-mm isotropic voxels) of the center-of-gravity of the superior colliculus-amygala overlap streamline of human participants in x and z coordinates in a series of eight consecutive slices in the y-plane in left and right hemisphere, and means for both hemispheres

Anterior/Posterior	Medial/Lateral		Superior/Inferior			
*y*-Plane Slice	Right *x*	Left *x*	Right *z*	Left *z*	Mean *x*	Mean *z*
51	1.58	0.87	0.82	1.08	1.23	0.95
52	0.69	0.83	0.79	1.11	0.76	0.95
53	0.43	0.86	0.72	1.05	0.65	0.89
54	0.39	0.87	0.69	0.91	0.63	0.80
55	0.51	0.83	0.67	0.95	0.67	0.81
56	0.61	0.75	0.66	1.01	0.68	0.84
57	0.59	0.84	0.61	1.23	0.71	0.92
58	0.64	1.03	0.90	1.01	0.84	0.95
Mean	0.68	0.86	0.73	1.04	0.77	0.89
SD	0.38	0.08	0.10	0.10	0.20	0.06

A similar approach was used to estimate intersubject variability in the monkey data. We analyzed a series of seven coronal slices covering a segment of the streamline that ran perpendicular to the coronal plane. Table 2 reports the variability (in the form of standard deviation) of the in-plane CoG across all subjects (omitting the hemispheres where no streamline was observed). There was greater variability in the positioning of the streamlines identified in monkeys compared with those identified in humans (standard deviation in monkeys: ∼2–3 mm, vs. 1–2 mm in humans), but, overall, the trajectories of the streamlines in both species were consistent across participant/subject.

**Table 2.** Standard deviation (in 0.5-mm isotropic voxels) of the center-of-gravity of the superior colliculus-amygala overlap streamline of monkey subjects in x and z coordinates in a series of seven consecutive slices in the y-plane in left and right hemisphere, and means for both hemispheres

Anterior/Posterior	Medial/Lateral		Superior/Inferior			
*y*-Plane Slice	Right *x*	Left *x*	Right *z*	Left *z*	Mean *x*	Mean *z*
94	3.6	0.9	2.7	2.2	2.3	2.5
96	3.4	0.8	4.5	2.2	2.1	3.4
98	3.5	1.4	4.1	1.9	2.5	3.0
100	3.2	1.6	3.7	1.6	2.4	2.6
102	3.3	1.7	4.1	1.7	2.5	2.9
104	3.2	2.2	4.0	2.2	2.7	3.1
106	3.1	2.4	4.3	2.4	2.7	3.3
Mean	3.3	1.6	3.9	2.0	2.4	3.0
SD	0.2	0.6	0.6	0.3	0.2	0.3

### The Stria Terminalis and Its Topographic Relation to the Connections between the SC and the Amygdala

**Fig. 8.**
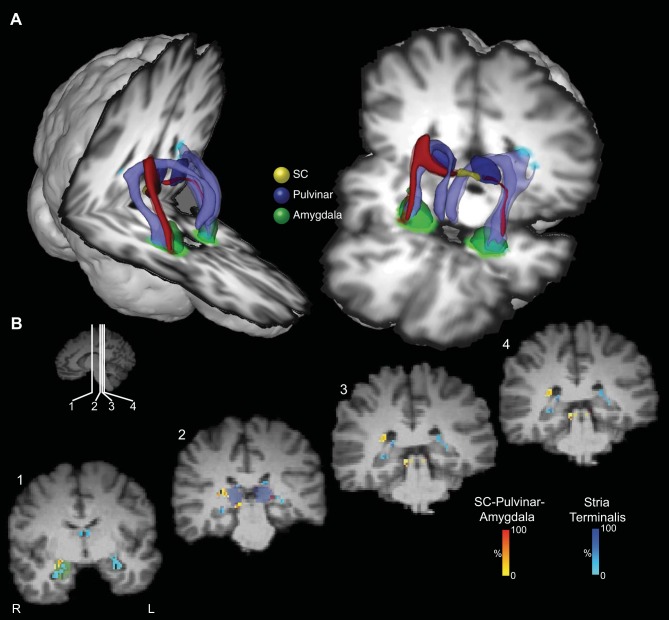
Location of the putative tract linking the SC and the amygdala relative to the stria terminalis in the human brain. *A*: 3D reconstructions of the SC-pulvinar-amygdala tract (shown in red) and the stria terminalis (shown in purple). Tracts have been thresholded by 10% (see methods for details). Unthresholded data are shown as transparency. The individual structures and tracts have been expanded in size for visualization purposes only (SC: 4 mm; amygdala and pulvinar: 2 mm; tract: 2 mm). *B*: coronal sections showing the location of the tract relative to the stria terminalis. The probabilistic data for both the SC-pulvinar-amygdala tract and the stria terminalis are presented unthresholded as a percentage of the total number of traces linking the starting and termination masks.

**Fig. 9.**
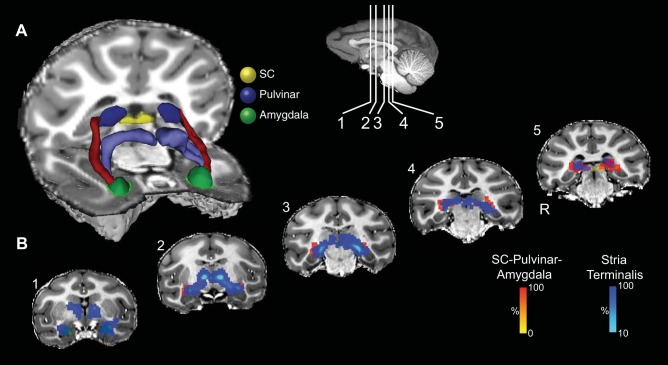
Location of the putative tract linking the SC and the amygdala relative to the stria terminalis in the monkey brain. *A*: 3D reconstruction of the SC-pulvinar-amygdala tract (shown in red) and the stria terminalis (shown in purple). Tracts have been thresholded by 10% (see methods for details). *B*: coronal sections showing the location of the tract relative to the stria terminalis. The probabilistic data for both the SC-pulvinar-amygdala tract and the stria terminalis are presented unthresholded as a percentage of the total number of traces linking the starting and termination masks.

Figures 8 and 9 show the projection of the stria terminalis (in pale blue) and its relationship to the connections between the SC and amygdala in a representative human participant (first group, Fig. 8) and monkey (Fig. 9). Similar topographies of the stria terminalis were seen in both hemispheres of all seven human participants in the first group in whom tracts between the SC to the amygdala via the pulvinar were identified, as well as in all but one hemisphere in the monkey subjects in whom SC-amygdala tracts were identified. In humans, the stria terminalis emerges from the amygdala and traverses above the temporal horn of the lateral ventricle and follows the lateral border of the tail of the caudate nucleus to pass between the body of the caudate nucleus and the border of the anterior thalamic nucleus before descending through the diencephalon to the region of the bed nucleus of the stria terminalis. In monkeys, the trajectory of the stria terminalis has a topography similar to humans.

The stria terminalis was also dissected in both hemispheres of all participants in the second human group, using the same method as for the first group. CoG measures of the stria terminalis in medial-lateral (*x*) and SI (*z*) coordinates was also computed in the coronal plane, for the same seven slices in the same segment above the temporal horn as described above for the analysis of variability of the SC-amygdala streamline. The CoG for the SC-amygdala and the stria terminalis streamlines for each of the eight coronal slices was submitted to separate ANOVAs for left-right (LR) (*x*) and SI (*z*) coordinates in each (left and right) hemisphere with participant as a between-subject factor. There was a reliable difference between the CoG of the two streamlines in both LR and SI planes in both hemispheres: left *X* mean coordinate *F*(1,11) = 768, *P* < 0.001; left *Z* mean coordinate *F*(1,11) = 246, *P* < 0.001; right mean *X* coordinate *F*(1,11) = 803, *P* < 0.001; right mean *Y* coordinate *F*(1,11) = 848, *P* < 0.001. The SC-amygdala streamline traverses above the temporal horn just medial to the stria terminalis [left hemisphere (mean 5.4 mm, SE 0.44 mm), right hemisphere (mean 7 mm, SE 0.44 mm)]. It passes dorsal to the stria terminalis [left hemisphere (mean 2.6 mm, SE 0.3 mm), right hemisphere (mean 3.6 mm, SE 0.2 mm)].

A similar analysis was performed on the monkey data, comparing the CoG measures for the SC-amygdala pathway and the stria terminalis across the seven coronal slices where the two streamlines paralleled each other as they traversed above the temporal horn. As in the human dataset, separate ANOVAs (excluding hemispheres where no streamline was observed for the SC-amygdala or stria terminalis) revealed a reliable difference in the CoG of the two streamlines in the LR and planes in both hemispheres: left *X* mean coordinate *F*(1,7) = 211, *P* < 0.001; right mean *X* coordinate *F*(1,7) = 267, *P* < 0.001. The SC-amygdala streamline traverses above the temporal horn lateral to the stria terminalis [left hemisphere (mean 2.5 mm, SE 0.33 mm), right hemisphere (mean 2.4 mm, SE 0.5 mm)]. There was no difference in the SI (*z*) plane between the two streamlines in either hemisphere.

### Analysis of the Integrity of Connections between the SC and Amygdala in Patients with Pulvinar Lesions

Evidence supporting the existence of a subcortical pathway from the pulvinar to the amygdala has been provided by single case studies of neurological patients. For example, cortically blind patients with visual cortex lesions may nonetheless demonstrate some ability to respond to emotional stimuli (Pegna et al. 2005). Neuroimaging (Dolan et al. 2001) and electrophysiological (Andino et al. 2009) evidence of amygdala activation by emotional stimuli in these patients is also consistent with a subcortical route.

The present study was motivated in part by the work of Ward and colleagues, who demonstrated, in single case studies, that unilateral medial pulvinar lesions delayed the effects of threatening contralesional visual stimuli (Ward et al. 2005) and, in another patient, impaired discrimination of fearful facial expressions in the contralesional field (Ward et al. 2007). In the latter study, three other patients with pulvinar lesions performed normally. Showing contralesional deficits in patients with lesions in this putative pathway, and sparing of function in other patients with pulvinar lesions in which the pathway is likely unaffected, would constitute strong evidence supporting the existence of a colliculo-thalamic-amygdala.

Unfortunately, neither of the patients who had deficits reported by Ward and colleagues are still available for scanning. However, diffusion-weighted imaging was obtained in one of the patients (DG) who had no contralesional deficit (Ward et al. 2007). The scanning protocols and virtual dissection methods were the same as those used for the first human group reported above.

**Fig. 10.**
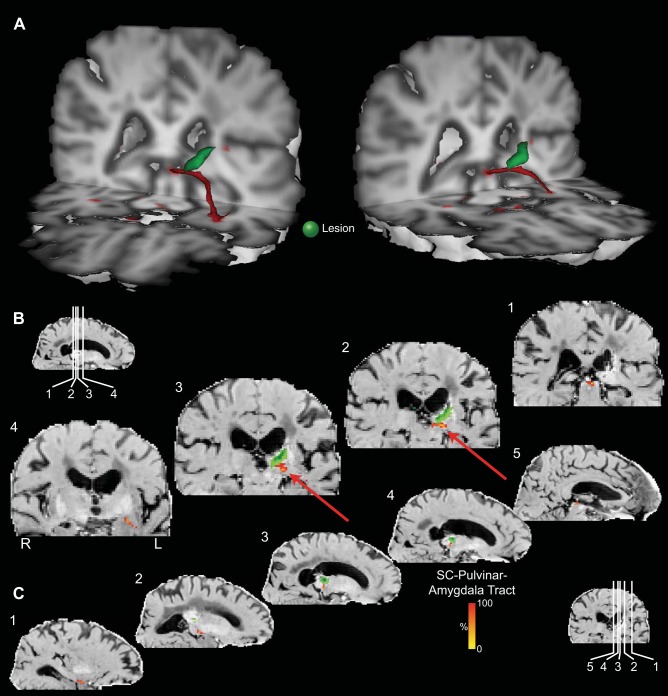
Probabilistic tractography between the SC and the amygdala via the pulvinar in patient DG. *A*: 3D reconstructions of the tract (shown in red) in relation to the site of the lesion (shown in green). Coronal (*B*) and sagittal (*C*) sections show the location of the tract relative to the lesion (green). Data are presented on a subtraction of the T2-weighted image [SO image generated by DTIfit from the FSL FDT Diffusion Toolbox] from the mean diffusivity image (MD image generated by DTIfit from the FSL FDT Diffusion Toolbox). This image highlights the lesion as a hypointense, cystic cavity in the pulvinar (masked with translucent green), surrounded by tissue with hemosiderin iron staining (high signal) from the previous hemorrhage. The probabilistic data are presented unthresholded (and actual size) as a percentage of the total number of traces linking the SC and amygdala (that pass through the pulvinar).

Figure 10 shows that the streamline between the SC, pulvinar, and amygdala in patient DG was intact in both hemispheres and was not interrupted by the lesion (highlighted with a green mask) in the left pulvinar. Note that the streamline passes through pulvinar tissue with hemosiderin iron staining (indicated by arrows in Fig. 10, *B* and *C*). Seen as high signal on these images, this is tissue that is functionally intact, but stained with iron containing hemosiderin residue in tissues surrounding the lesion caused by the cerebral hemorrhage.

In four other patients with pulvinar lesions for whom DTI data are not available, we generated individual masks for the lesions [drawn on T1-weighted structural images and normalized to Montreal Neurological Institute (MNI) space] and sought to determine whether the lesion would likely have interrupted the streamline. A compound streamline, normalized to MNI space, from each hemisphere of the 12 healthy participants in the second group was generated (Fig. 2*C*), and the topography of the streamline in relation to the lesion was examined. The compound streamline was generated by adding the streamlines for all 12 participants in each hemisphere and thresholding it to show only those voxels that were common to the streamlines of at least 8 of the 12 participants.

**Fig. 11.**
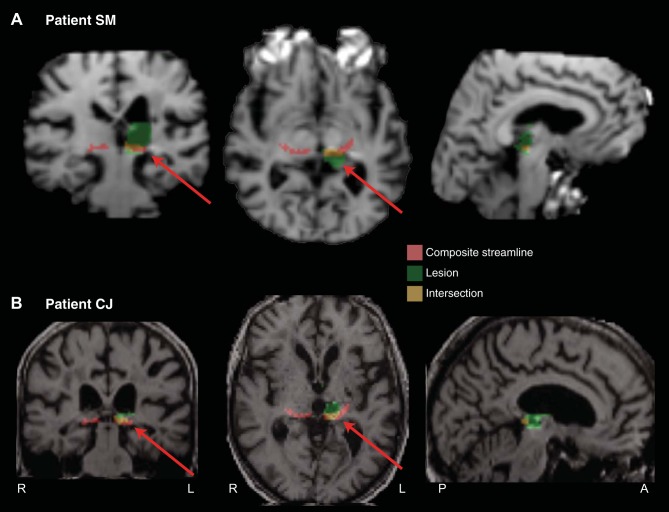
Coronal, axial, and sagittal slices for two patients who exhibited a contralesional behavioral deficit in the emotional recognition tasks [Ward et al. 2005 (patient SM; *A*) and 2007 (patient CJ; *B*)].

Figure 11*A* shows the lesion of patient SM who showed delayed threat detection in the contralesional field (Ward et al. 2005). It is clear that the streamline connecting the SC with the amygdala would likely have been interrupted in his damaged hemisphere (intersection between composite streamline and lesion indicated by coloration and arrows). Figure 11*B* shows the lesion of patient CJ who was at chance in identifying fearful faces in the contralesional visual field. This figure also shows that the streamline connecting the SC with the amygdala would have been interrupted in his damaged hemisphere.

**Fig. 12.**
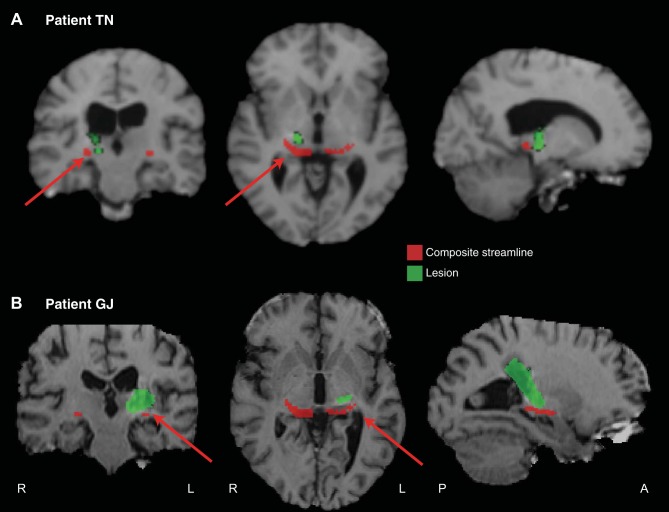
Coronal, axial, and sagittal slices for two patients [TN (*A*) and GJ (*B*)] who, like patient DG, exhibited preserved abilities following pulvinar damage (Ward et al. 2007).

Figure 12, *A* and *B*, shows the lesions of the two other patients (TN and GJ) who (like DG) had no deficit in identifying emotional facial expressions. This figure suggests that the lesion would not have interrupted the streamline in the damaged hemisphere. Thus a behavioral deficit was observed in all cases where the streamline would likely have been disrupted, and no deficit when the streamline would presumably have been left intact.

## DISCUSSION

In both hemispheres of 19 of 20 healthy human participants (7 out of 8 in the first group, and in all 12 people in the second group), and in 7 of 8 macaque monkeys, probabilistic DTI tractography demonstrated potential anatomical connectivity between the SC and the amygdala through a streamline traversing the pulvinar. We also demonstrate, for the first time in any species, the topography of the connections of a collo-thalamic projection between the thalamus and the amygdala. These connections ascend from the SC, pass through the medial pulvinar to the pole of the pulvinar, and then descend, turning laterally and rostrally, to pass through the lateral pulvinar. Exiting the pulvinar, the streamline traverses above the temporal horn of the lateral ventricle, where it is positioned dorsal and medial to the stria terminalis, and, terminally, connects to the lateral aspect of the amygdala.

The current observations in humans are consistent with a recent study (Tamietto et al. 2012) that employed deterministic tractography demonstrating potential connectivity between the SC and the amygdala. In that study, separate tracts were generated from the SC to the pulvinar, and from the pulvinar to the amygdala. In addition, that study also demonstrated connectivity between SC and amygdala as an isolated streamline by using both amygdala and SC as seed masks. Here we have also demonstrated connectivity between the SC and amygdala generated as a single, isolated streamline.

The results of Tamietto et al. (2012) did not describe the precise topology of the connections between SC and amygdala, nor specify the anatomical relationship between these connections and other anatomical structures such as the stria terminalis, and so we cannot confirm that the connections reported in the present research represent the same anatomical structures reported by Tamietto and colleagues. However, their Supplemental Fig. S3 shows a streamline traversing over the temporal horn. The same figure clearly shows the stria terminalis, however, and so it is not possible to unambiguously assign the streamline traversing superior to the temporal horn, shown in Fig. S3, to the SC-pulvinar-amygdala pathway.

Using two novel approaches to the problem, we were able to show that the connections from pulvinar to amygdala are independent from the stria terminalis and cannot be an artifact of an intersection with it. First, we used the stria terminalis as an exclusion mask in the dissection of the SC-pulvinar-amygdala streamline. We also used a CoG analysis to demonstrate that the segments of these pathways that traverse above the temporal horn of the lateral ventricle are anatomically distinct.

Colliculo-thalamic projections to cortex send collaterals to the amygdala. In the rat (Linke et al. 1999), this collateral pathway projects from the SC via the suprageniculate nucleus (one of the posterior thalamic nuclei, as rodents do not have a distinguishable pulvinar) to the lateral amygdala. In the tree shrew (*Tupaia belangeri*), a species considered to be a prototypical primate (Fan et al. 2013), the SC projects to the pulvinar, with topographic efferents from the SC projecting to the dorsal pulvinar nucleus, which projects efferents to the amygdala (Day-Brown et al. 2010). In macaque monkeys, Benevento and Standage (1983) demonstrated that SC efferents project to inferior, medial and lateral pulvinar nuclei.

However, unlike the tree shrew, tracer studies in monkeys have not, to date, identified projections to the amygdala from pulvinar nuclei that receive afferents from the superficial layers of the SC. Benevento and Standage (1983) found that only the inferior and lateral pulvinar nuclei received afferents from the superficial layers of the SC, whereas the medial thalamic nucleus receives collicular afferents only from the deep layers. Since efferents from the pulvinar to the lateral amygdala have been demonstrated from the medial pulvinar nucleus in the macaque monkey, but not from the inferior or lateral pulvinar, it is not clear how coarse visual signals conveying threat could be relayed through the pulvinar to the amygdala (Pessoa 2005). Although tracer studies have not, to date, demonstrated such anatomical connectivity, it has nevertheless been shown recently that neurons in both medial and lateral pulvinar nuclei respond with short latencies to threatening stimuli; in the case of pictures of snakes, some neurons respond with latencies of <60 ms (Van Le et al. 2013). Moreover, two recent studies have reported that single cells in the pulvinar of macaque monkeys respond differentially to human facial expressions (Maior et al. 2010), and that the SC responds to faces and face-like stimuli (Nguyen et al. 2014).

Using a retrograde tracer (horseradish peroxidase) in rhesus macaque monkeys, Aggleton et al. (1987) also showed that the medial pulvinar was lightly labeled after injections in the lateral amygdala. Jones and Burton (1976) reported medial pulvinar efferents that sent collaterals to the anterior, lateral amygdala in both squirrel monkeys and macaques. They did not examine whether this region of the pulvinar received afferents from the SC. Their paper included a figure reconstructing tracer topography (Fig. 1) only in one squirrel monkey, and so we cannot readily compare their findings with those reported here in macaques. Their figure showed an injection site near the border between lateral and medial pulvinar. It did not show slices between the injection site and the pulvinar and the amygdala. It demonstrated fibers entering the lateral amygdala from the external capsule. However, it also demonstrates tracer above the temporal horn, medial to the tail of the caudate nucleus.

Probabilistic DTI tractography reflects continuity of anisotropic diffusion and can be used to infer the path of axons. It provides no information about either the direction of axonal transmission or the presence of synapses. Our findings therefore shed no light on specific neural circuitry. They are equally consistent with a monosynaptic pathway between the SC and the amygdala connecting in the medial pulvinar, as well as a multisynaptic pathway that might include one or more additional synapse in the pulvinar. Indeed, probabilistic DTI tractography cannot be taken as definitive evidence for either pathway.

It must be emphasized that, while the current study and that of Tamietto et al. (2012) show connections between the pulvinar and amygdala, and connections between the SC and those voxels within the pulvinar through which a streamline passes to the amygdala, it is not possible with tractography to show that there are connections between the retino-recipient superficial layers of the colliculus and pulvinar neurons that then project to the amygdala. The streamline demonstrated in the figures reported here traverses the medial and lateral pulvinar and may consist of voxels containing both axons to the medial pulvinar from the deep layers of the SC and axons from the superficial layers of the SC projecting to the lateral pulvinar. Moreover, it is not currently possible to define homology between the dorsal pulvinar nucleus of the tree shrew that receives afferents from retino-recipient SC and projects to the amygdala, with any corresponding pulvinar nuclei in macaques.

Confirmation that the virtual dissections reported here correspond to a functional anatomical pathway transmitting visual information signaling threat will require further investigation. We propose that DTI tractography can provide a valuable tool for testing this hypothesis through converging studies in humans and monkeys.

To return to issues raised in our introduction, we have documented the potential of a subcortical pathway that is likely to be involved in transmitting visual threat. We do not conclude that this subcortical path is “faster” than a geniculostriate path, but we suggest activation of the amygdala will jointly reflect the input of cortical and subcortical routes. Disruption to a subcortical pathway would therefore be expected to slow or degrade amygdala activation relative to fully intact pathways.

We also provide preliminary evidence, as a proof of concept, that tractography can be a useful lesion analysis tool in future neuropsychological investigations of this putative subcortical visual pathway for transmitting visual threat. We have also seen how neuropsychological results can, in principle, address the functional role of pathways found through tractography. In this case, two patients with fear and threat processing deficits were retrospectively found to have lesions extending into the putative pathway, while for three other patients without fear deficits, it was retrospectively found that their lesions, while similar in many respects, did not extend into the pathway.

These preliminary observations in patients with pulvinar lesions converge with neuropsychological evidence in patients with blindsight to support the case for a subcortical pathway from the SC to the amygdala that functions in processing emotional salience. While observations in patients with blindsight provide evidence that emotionally valenced visual stimuli can activate the amygdala without involvement of primary visual cortex, they do not show that such stimuli activating the pulvinar are necessarily transmitted to the amygdala via a subcortical pathway. The pulvinar connects to many areas of cortex, and some of these could transmit signals to the amygdala (McDonald 1998). The current analyses in patients with pulvinar lesions provide evidence that the area of pulvinar that processes emotionally valenced stimuli is likely connected to the amygdala by a subcortical pathway that traverses above the temporal horn. However, while these analyses also show connectivity of the same area of the pulvinar to the SC, they do not provide direct evidence that these SC-pulvinar connections receive visual signals from retino-recipient layers of the SC that are transmitted to the amygdala. Indeed, in their analyses of connectivity strength, Tamietto et al. (2012) showed that, in the lesioned hemisphere of hemianopic patient GY, connectivity was stronger than controls between the pulvinar and the amygdala which was not the case for connections between the SC and the pulvinar.

More definitive evidence that these subcortical connections function in transmitting emotion signals to the amygdala will require direct physiological studies targeting the different stages of the subcortical pathway. This could best be achieved in the monkey using invasive techniques, including anatomical tracer studies and/or sophisticated electrophysiological recording studies. Our results could then be used as supporting evidence to extrapolate back to the human brain.

## GRANTS

This research was supported by the Medical Research Council (MRC) UK/MRC Intramural Programme (MRC Cognition and Brain Sciences Unit), and MRC Career Development Award G0800329 to A. S. Mitchell.

## DISCLOSURES

No conflicts of interest, financial or otherwise, are declared by the author(s).

## AUTHOR CONTRIBUTIONS

Author contributions: R.D.R., R.W., A.S.M., and A.H.B. conception and design of research; R.D.R., K.K., P.M., and A.H.B. performed experiments; R.D.R., K.K., J.H.B., P.M., and A.H.B. analyzed data; R.D.R., K.K., P.M., R.W., A.S.M., and A.H.B. interpreted results of experiments; R.D.R., K.K., and A.H.B. prepared figures; R.D.R. and A.H.B. drafted manuscript; R.D.R., K.K., J.H.B., P.M., R.W., A.S.M., and A.H.B. edited and revised manuscript; R.D.R., K.K., J.H.B., P.M., R.W., A.S.M., and A.H.B. approved final version of manuscript.
